# Audiological characteristics and cochlear implant outcome in children with cochlear nerve deficiency

**DOI:** 10.3389/fneur.2022.1080381

**Published:** 2022-12-22

**Authors:** Cuncun Ren, Ying Lin, Zhuo Xu, Xiaoqin Fan, Xinyu Zhang, Dingjun Zha

**Affiliations:** Department of Otolaryngology, Head and Neck Surgery, Xijing Hospital, PLA Air Force Military Medical University, Xi'an, China

**Keywords:** cochlea nerve deficiency, cochlear implant, pediatric, audiological characteristic, auditory and speech ability

## Abstract

**Objectives:**

This study aimed to examine the audiological characteristics and validity of predicting outcomes of cochlear implants (CIs) in children with cochlear nerve deficiency (CND) based on the internal auditory meatus (IAM) nerve grading system.

**Methods:**

The audiological characteristics of 188 ears in 105 children diagnosed with CND were analyzed based on the IAM nerve grading system. In addition, 42 children with CND who underwent CI were also divided into four groups based on the system, and their auditory and speech performance at baseline (preoperative) and 6, 12, and 24 months after CI were analyzed and compared with those of the control group (*n* = 24) with a normal cochlear nerve (CN) and CI.

**Results:**

The audiological test results showed no significant differences among the four CND groups in terms of elicited rates of distortion product otoacoustic emission (DPOAE) (*p* = 1.000), auditory brainstem response (ABR) (*p* = 0.611), and cochlear microphonic (CM) (*p* = 0.167). Hearing in the CND IV group was significantly better than that in the CND I group (*p* < 0.05). In children with CI, the auditory and speech performance of the control group was significantly higher than all CND groups from 6 to 24 months (*p* < 0.05) and 12 to 24 months (*p* < 0.05), respectively. Meanwhile, there were no significant differences between each pair group in the four CND groups (*p* > 0.05).

**Conclusion:**

Children with CND, including those in whom the CN was not visualized by MRI, can benefit from CI. Additionally, the IAM nerve grading system could not predict the outcomes of CI in children with CND.

## 1. Introduction

Cochlear nerve deficiency (CND) is defined as a small or absent cochlear branch of the vestibulocochlear nerve; the clinical manifestation of this condition is sensorineural hearing loss (SNHL) with unclear etiology and pathogenesis. The incidence of CND in patients with congenital SNHL ranges from 2.5 to 21.2% (1, 2). Oblique sagittal high-resolution magnetic resonance imaging (MRI) is now routinely performed in children with SNHL to diagnose CND.

However, the current MRI resolution has limitations, making the differentiation of the CN from other nerves in the internal acoustic meatus (IAM) difficult (3). Cochlear implants (CIs) in patients with CND remain controversial as some studies involving CI recipients with CND have reported very poor results (4), while others have reported limited speech detection and discrimination. Meanwhile, some studies have reported higher levels of auditory performance following CI (5–10). Researchers have proposed a new IAM nerve grading system and a CN classification system based on MRI findings of nerves within the IAM and the size of the CN, which is as follows: grades 0–III indicated zero, one, two, and three nerve bundles observed in the IAM, respectively (aplasia); grade IV, four nerve bundles in the IAM with a hypoplastic CN (hypoplasia); and grade V, four nerve bundles in the IAM with a normal-sized CN and normal position of the nerves (normal). The results of a study showed that patients with IAM grade IV had higher auditory performance than those with grades 0–III (11). Although recent studies have evaluated the validity of this CN classification system to predict outcomes of CI in patients with CND, the results have been varied, and few studies have reported longitudinal results on auditory and speech performance after CI (12, 13); this is important because children with CND might have delayed auditory and speech development.

This study aimed to analyze the audiological characteristics in children with CND and to investigate the auditory and speech performance of children with CND over time after CI. In addition, we aimed to answer the question “Does the IAM nerve grading system predict CI outcomes in children with CND?” This study is useful for clinicians who are contemplating cochlear implantation in patients with CND, especially those in whom the CN was not visualized by MRI.

## 2. Materials and methods

### 2.1. Subjects

This study was approved by the Ethics Committee of our hospital, and 105 children (45 boys and 60 girls; mean age at diagnosis, 3.00 ± 1.88 years; range, 0.52–8.11 years) diagnosed with CND based on temporal bone computed tomography (CT), and MRI scan was included for audiological characteristic analysis. Among them, 83 children had bilateral CND, and 22 children had unilateral CND. Consequently, the audiological characteristics were analyzed in 188 ears.

To determine the outcomes of CI in children with CND, we screened subjects with unilateral CI for postoperative auditory and speech ability analysis. The inclusion criteria were as follows: prelingual profound SNHL; at least 2 years of having a CI; rehabilitation for at least 1 year using auditory–verbal therapy; and preoperative diagnosis of CND. The exclusion criteria were as follows: syndromic SNHL such as Usher syndrome, Waardenburg syndrome, or CHARGE syndrome; and other disabilities, such as cretinism and mental or developmental disorders. In total, 42 children were included in the analysis. The mean age at implantation was 2.8 ± 1.7 years (range, 0.7–8.2 years). A total of 24 unilateral CI recipients without inner ear malformations (IEMs) and CND were included in the control group (mean age, 2.3 ± 1.1 years; range, 0.9–4.9 years). The control and study groups were matched in terms of implantation age, CI use, and rehabilitation to minimize confounding factors that could affect the outcomes of auditory and speech ability. All subjects underwent cochlear implantation at the ENT Department of Xijing Hospital. The demographic characteristics of the CI subjects are shown in Table 1.

**Table 1:** Clinical characteristics of CND groups and control group.

	**CND_I** **(*n* = 5)**	**CND_II** **(*n* = 6)**	**CND_III** **(*n* = 15)**	**CND_IV** **(*n* = 16)**	**Control group** **(*n* = 24)**
Age at implantation (yr)	2.47 ± 1.69	3.64 ± 1.84	2.65 ± 1.53	2.66 ± 1.36	2.33 ± 1.10
**Gender**, ***n***					
Male	0	3	2	9	10
Female	5	3	13	7	14
**Side of implantation**, ***n***					
left	2	3	3	6	4
Right		2	10	7	17
Bilateral	3	1	2	3	3
Period of HA use before CI (mo)	10.60 ± 9.39	12.40 ± 5.82	9.65 ± 6.42	11.03 ± 5.34	6.65 ± 5.83
**HA use after CI in the contralateral ear**, ***n***					
Yes	1	3	8	10	14
No	1	2	5	3	7
**Inner ear malformations**, ***n***					
Yes	2	2	8	3	0
No	3	3	5	10	24
**CI device**, ***n***					
MED-EL	4	4	11	8	15
Cochlear		1	2	6	4
Advanced bionics	1	1	1	2	3
Nurotron			1		2

CND, cochlear nerve deficiency; HA, hearing aid; CI, cochlear implant.

### 2.2. Radiological evaluation

Radiological evaluation of patients with CND was performed retrospectively using available CT and MRI images. All included patients were confirmed as having CND by HRCT and MRI. CT was mainly used to determine whether the IAM was stenotic and whether there were IEMs. MRI images, particularly parasagittal IAM views, provided precise information regarding the CN. Children with CND were divided into four groups according to the IAM nerve grading system (11). All imaging findings were assessed by a senior experienced radiologist. The radiographic characteristics of CI subjects are shown in Table 1.

### 2.3. Audiological evaluation

All patients underwent audiological assessments, including neonatal hearing screening, subjective audiometry (pure-tone audiometry or behavioral audiometry), acoustic immittance, distortion product otoacoustic emission (DPOAE), auditory brainstem response (ABR), cochlear microphonic (CM), and auditory steady-state response (ASSR) testing. The hearing thresholds were calculated by the average hearing thresholds at 0.5, 1, 2, and 4 kHz; if there was no response at the maximum output of the audiometer, the threshold was considered 5 dB greater than the maximum output for the purpose of averaging. Notably, 13 subjects (24 ears) failed to undergo behavioral audiometry due to young age; instead, ASSR was used to determine the hearing thresholds.

### 2.4. Postoperative auditory and speech evaluation

Postoperative auditory performance was evaluated using several scales. The Categories of Auditory Performance (CAP) scale classifies auditory ability with scores ranging from 0 to 7. The (Infant–Toddler)-Meaningful Auditory Integration Scale (MAIS for ages >3 years, IT-MAIS for ages <3 years) comprises 10 items, and the frequency of each item is categorized as never (0), seldom (1), sometimes (2), often (3), and always (4); the final score is calculated as the sum of the scores of the 10 items. Speech performance was evaluated using both the Speech Intelligibility Rating (SIR) scale, which classifies speech ability with scores ranging from 1 to 5, and the Meaningful Use of Speech Scale (MUSS), wherein the final score is calculated as the sum of the scores of 10 items. The evaluations were performed on the five groups of CI recipients at baseline (preoperative) and 6, 12, and 24 months after CI.

### 2.5. Data analysis

All statistical analyses were conducted using SPSS 22.0 (IBM, Armonk, NY, USA). The chi-square test and Fisher's exact test were performed to analyze the audiological test results for the four CND groups, and the Bonferroni test was used for multiple comparisons. The repeated measures analysis of variance was used to analyze the scores of CAP, ITMAIS, SIR, and MUSS. Mauchly's test was used to assess the sphericity assumption, and *F*-values were adjusted by the Greenhouse–Geisser correction if the sphericity assumption had been violated. When there was a significant difference in the interaction group–time, analysis of variance was used to compare the mean scores between two groups at each time point, and Scheffe's test was used for pairwise comparisons. Data with missing values were excluded from the analyses. The *p* < 0.05 were considered statistically significant in all analyses.

## 3. Results

### 3.1. Audiological characteristics of children with CND

Neonatal hearing screening results were obtained in 56 of 105 children with CND, and 36 of 112 (32%) ears passed the first screening. We further analyzed the age at the diagnosis of hearing loss in children who had different neonatal hearing screening results. Unsurprisingly, we found that children who failed the hearing screening in both ears were diagnosed at a mean age of 4.36 ± 5.24 months, which was significantly earlier than the mean age of 18.76 ± 10.33 months in children who passed the hearing screening in at least one ear (*p* < 0.001).

Table 2 shows the percentage of ears with CND grades I–IV among the 188 ears, the ratio of ears with IEMs, elicitation rates of DPOAE, CM, and ABR, and the percentage of ears with a hearing threshold of <120 dB HL.

**Table 2:** Percentages of ears with inner ear malformations and elicitation rates of DPOAE, ABR, CM, and hearing threshold of <120 dB HL grouped by the IAM nerve grading system.

**CN classification**	**Inner ear malformation**	**DPOAE**	**ABR**	**CM**	**Hearing threshold < 120 dB HL**
Grade I	41%(7/17)^a^, ^b^	6%(1/17)	0%(0)	27%(3/11)	41% (7/17)^c^
Grade II	52%(11/21)^a^, ^b^	9%(2/21)	5%(1/21)	70%(7/10)	52% (11/21)^c^, ^d^
Grade III	63%(40/64)^b^	8%(5/64)	8%(5/64)	34%(12/35)	47% (30/64)^c^, ^d^
Grade IV	38%(33/86)^a^	7%(6/86)	9%(8/86)	37%(10/27)	67% (58/86)^d^
χ^2^ value	9.046	0.232	2.029	5.060	8.365
*p*-value	0.028	1.000	0.611	0.167	0.038
Total	48% (91/188)	7% (14/188)	9% (16/188)	39% (32/82)	56% (106/188)

a, b, c, d There was no significant difference between the groups, which is marked by the same letter.

CN, cochlear nerve; DPOAE, distortion product otoacoustic emission; CM, cochlear microphonic; ABR, auditory brainstem response; IAM, internal acoustic meatus.

Distortion product otoacoustic emission responses were elicited in 7% of the ears (14/188), CM responses were elicited in 39% of the ears (32/82), and ABRs were elicited in 9% of the ears (16/188). There was no significant difference between the four CND groups (χ^2^ = 0.232, *p* = 1.000 for DPOAE; χ^2^ = 0.029, *p* = 0.611 for ABR; χ^2^ = 5.060, *p* = 0.167 for CM). Moreover, the elicitation rate of OAE was significantly lower than that of CM (*p* < 0.05). The percentage of ears with a hearing threshold of < 120 dB HL was 56% (106/188). There was a significant difference in the overall percentage between the four CND groups (χ^2^ = 8.365, *p* = 0.038), and multiple comparisons showed that the percentage of the CND grade IV group was significantly higher than that of the CND grade I group (*p* < 0.05).

### 3.2. CI outcomes in children with CND

**Figure 1:**
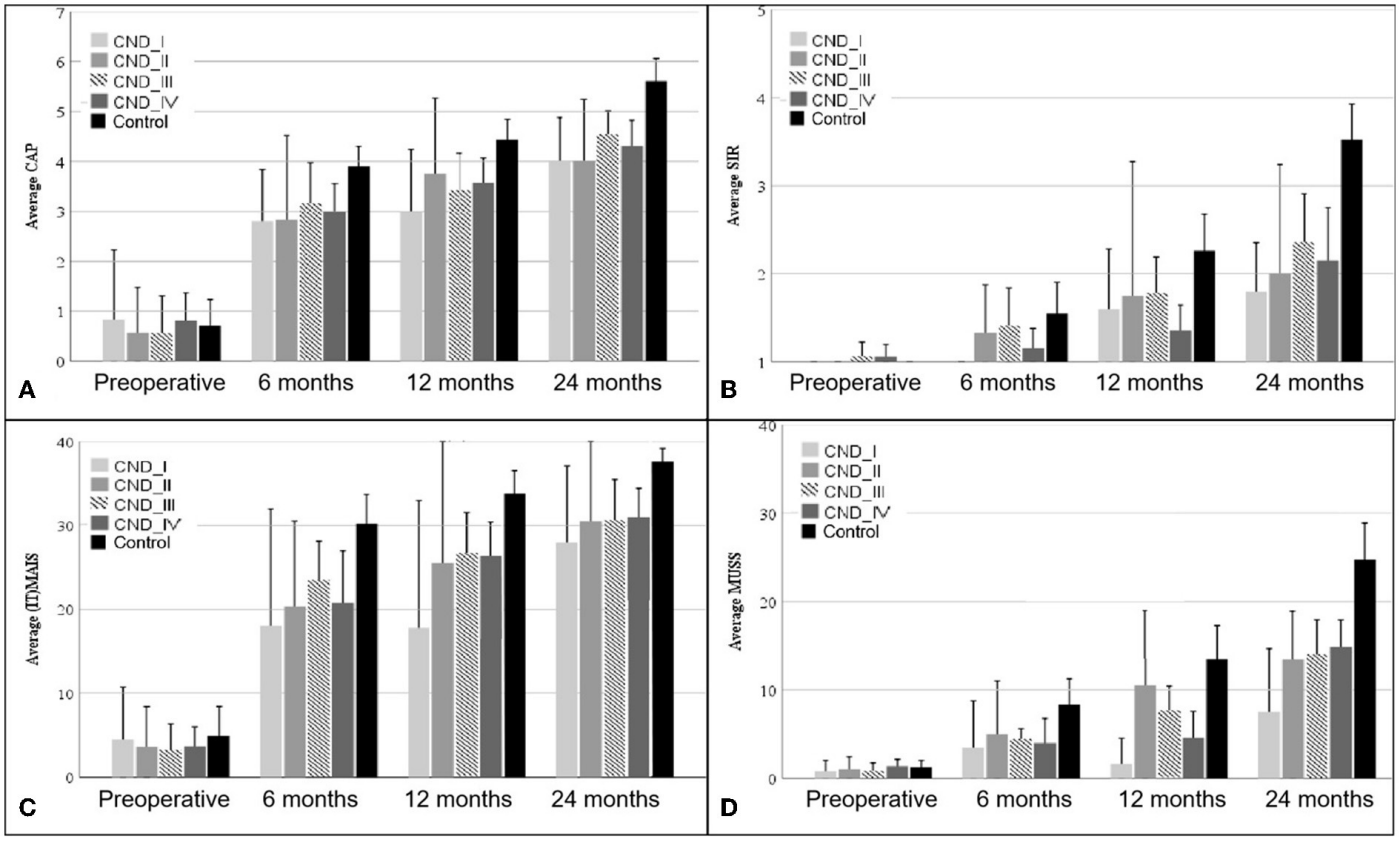
CAP **(A)**, SIR **(B)**, (IT)-MAIS **(C)**, and MUSS **(D)** scores of patients in the four CND groups and the control group at baseline (preoperative) and 6, 12, and 24 months after CI. The error bars show the standard error. CND, cochlear nerve deficiency; CAP, Categories of Auditory Performance; (IT)-MAIS, (Infant–Toddler)-Meaningful Auditory Integration Scale; SIR, Speech Intelligibility Rating; MUSS, Meaningful Use of Speech Scale.

Figure 1 shows the mean and error bars of the CAP, (IT)-MAIS, SIR, and MUSS scores of children in the four CND groups and the control group at baseline and at 6, 12, and 24 months after CI. Table 3 shows the analysis of variance results of the four scales at each time point in the four CND groups and the control group.

**Table 3:** Analysis of variance results of four scales at each time point.

	**CAP**		**(IT)MAIS**		**SIR**		**MUSS**	
	**F**	**P**	**F**	**P**	**F**	**P**	**F**	**P**
Preoperative	0.372	0.828	0.179	0.948	0.599	0.665	0.224	0.924
6 Months	2.827	0.034^*^	4.183	0.005^*^	1.421	0.240	2.115	0.094
12 Months	4.961	0.002 ^*^	6.211	< 0.001^*^	3.269	0.018^*^	4.096	0.006^*^
24 Months	10.687	< 0.001^*^	6.597	< 0.001^*^	7.722	< 0.001^*^	7.626	< 0.001^*^

* There is a significant difference.

CND, cochlear nerve deficiency; CAP, Categories of Auditory Performance; (IT)-MAIS, (Infant-Toddler)-Meaningful Auditory Integration Scale; SIR, Speech Intelligibility Rating; MUSS, Meaningful Use of Speech Scale.

#### 3.2.1. Auditory performance

The Categories of Auditory Performance scores of the four CND groups and the control group at different time points after CI are shown in Figure 1A. The MAIS/IT-MAIS results are shown in Figure 1C. Both scale results of Mauchly's test of sphericity showed a violated assumption (*p* < 0.001); thus, *F*-values were adjusted by the Greenhouse–Geisser correction. There was a significant difference in the interaction group–time (*F* = 2.312, *p* = 0.028 for CAP; *F* = 2.743, *p* = 0.020 for MAIS/IT-MAIS); therefore, scores of the four CND groups and the control group at each time point were calculated separately. At baseline, there was no significant difference between the five groups (*F* = 0.372, *p* = 0.828 for CAP; *F* = 0.179, *p* = 0.948 for MAIS/IT-MAIS). At 6 months, the results showed a significant difference between the five groups, and this difference persisted until 24 months (*F* = 2.827, *p* = 0.034 at 6 months; *F* = 4.961, *p* = 0.002 at 12 months; *F* = 10.687, *p* = < 0.001 at 24 months for CAP. *F* = 4.183, *p* = 0.005 at 6 months; *F* = 6.211, *p* < 0.001 at 12 months; *F* = 6.597, *p* < 0.001 at 24 months for MAIS/IT-MAIS). Pairwise analysis showed that the CAP scores of the control group were significantly higher than those of the four CND groups from 6 months to 24 months (*p* < 0.05), and that there was no significant difference between each paired group among the four CND groups at every time point (*p* > 0.05). Pairwise analysis showed that the MAIS/IT-MAIS scores of the control group were significantly higher than that of the CND I group (*p* = 0.02) and CND IV group (*p* = 0.005) at 12 months and all four CND groups at 24 months (*p* = 0.044, *p* = 0.048, *p* = 0.026, *p* = 0.002). There was no significant difference between the four CND groups at each time point (*p* > 0.05).

Table 4 shows the detailed CAP scores of each subject in the four CND groups and the control group at 24 months after CI. Percentages of patients who achieved CAP scores <3 (recognizing environmental sounds but not understanding speech words), 4–5 (understanding some words and common phrases), and 6–7 (understanding a conversation in person or on the telephone) are also listed. The difference between groups was significant (*p* < 0.001). The results of multiple comparisons showed that the percentage of patients in the control group who achieved higher CAP scores was significantly higher than that in the CN hypoplasia and CN aplasia groups (*p* < 0.001). Meanwhile, there was no significant difference between the CN hypoplasia and CN aplasia groups (*p* > 0.05).

**Table 4:** CAP scores based on CN classification in 43 CI recipients.

**CN classification**	**Aplasia**			**Hypoplasia**	**Normal**
	**CND_I** **(*n* = 5)**	**CND_II** **(*n* = 6)**	**CND_III** **(*n* = 15)**	**CND_IV** **(*n* = 16)**	**Control group** **(*n* = 24)**
CAP 0, no auditory perception	0	0	0	0	0
CAP 1, detects environmental sounds	0	0	0	0	0
CAP 2, responds to speech sounds	0	0	0	0	0
CAP 3, identifies environmental sounds	1	2	1	3	0
CAP 4, understands words	3	2	7	6	1
CAP 5, understands common phrases	1	2	8	7	9
CAP 6, understands a conversation	0	0	0	0	9
CAP 7, understands a conversation on the telephone	0	0	0	0	5
CAP 0–3	15.40% (4/26)^a^			18.75% (3/16)^a^	0%
CAP 4–5	84.6% (22/26)^b^			81.25% (13/16)^b^	41.70% (10/24)
CAP 6–7	0%			0%	58.30% (14/24)

a, b There was no significant difference between the groups, which is marked by the same letter.

CN, cochlear nerve; CAP, categories of auditory performance; CI, cochlear implant.

#### 3.2.2. Speech performance

The Speech Intelligibility Rating and MUSS scores are shown in Figures 1B, D. Both scale results of Mauchly's test of sphericity also showed a violated assumption (*p* = 0.004 for SIR; *p* < 0.001 for MUSS); therefore, the *F*-values were adjusted by the Greenhouse–Geisser correction. There was a significant difference in the interaction group–time (*F* = 3.398, *p* = 0.001 for SIR; *F* = 4.069, *p* < 0.001 for MUSS); therefore, scores for the four CND groups and the control group at each time point were calculated separately. At baseline, there was no significant difference among the five groups (*F* = 0.599, *p* = 0.665 for SIR; *F* = 0.224, *p* = 0.924 for MUSS). The results showed a significant difference between the five groups at 12 and 24 months (*F* = 3.269, *p* = 0.018 at 12 months; *F* = 7.722, *p* < 0.001 at 24 months for CAP; *F* = 4.096, *p* = 0.006 at 12 months; *F* = 7.626, *p* < 0.001 at 24 months for MUSS). Pairwise analysis showed that the CAP scores of the control group were significantly higher than those of the CND IV group (*p* = 0.025) at 12 months and all CND groups at 24 months (*p* = 0.040, *p* = 0.046, *p* = 0.022, *p* = 0.004). There was no significant difference between the four CND groups at each time point (*p* > 0.05). Pairwise analysis showed that the MUSS scores of the control group were significantly higher than those of the CND IV group (*p* = 0.043) at 12 months and all CND groups at 24 months (*p* = 0.031, *p* = 0.046, *p* = 0.039, *p* = 0.002). There was no significant difference between the four CND groups at each time point (*p* > 0.05).

## 4. Discussion

### 4.1. Audiological characteristics of ears with CND

The passing rate in the neonatal hearing screening in children with CND was 32%, indicating that if OAE is the only measure used for neonatal hearing screening, approximately one-third of children with CND will be undiagnosed; this would lead to a significantly delayed diagnosis of hearing loss in these children, which will result in delayed intervention and rehabilitation. This scenario highlights the importance of using a combination of OAE and auto ABR in neonatal hearing screening (14).

The elicitation rate of OAE was much lower than that of CM during audiological assessments in these children, which may be because CM is less susceptible to conduction factors than OAE when evaluating cochlear function (15). Therefore, if neither OAE responses nor ABRs are elicited, attention should also be paid to CM, which would aid in differentially diagnosing sensory and retrocochlear hearing loss.

Regarding hearing threshold in children with CND, the results revealed that nearly half of the ears with CND grades I to III presented with hearing (average hearing threshold < 120 dB HL), and approximately two-thirds of the ears with CND grade IV presented with hearing. The results indicate that although MRI is essential for confirming CND, it might not be possible to distinguish between hypoplasia and aplasia due to its limited resolution, which is consistent with the findings of some previous studies (16). Researchers have concluded that the CN not being visible on MRI may be due to the following reasons. First, artifacts that interfere with CN imaging may arise due to various reasons, including patient motion. Second, nearby structures, such as vascular loops and cerebellar crowding, may obscure the nerves. Third, CND is often associated with temporal bone malformations, such as stenosis of the IAC or a bony CN canal, leading to difficulty in visualizing the CN. Finally, in some cases, the CN may move against the wall of the IAC, may not be separated from the vestibulocochlear nerve, or may run outside the IAC. CN fibers may still be present even if the CN is not visible on the MRI (17).

A full audiological test battery that includes both subjective and objective tests should be performed to confirm the presence of hearing in patients with CND (18). Subjective tests are important even when no responses are observed during the other tests; therefore, it might be the only method that could provide information regarding the hearing status of children. Observable behavioral responses to pure-tone or speech stimulation, despite apparent CN aplasia on MRI, signify that the children may benefit from CI.

### 4.2. CI outcomes in children with CND

Cochlear implant in children with CND, especially those with CN aplasia, has been controversial. In theory, the benefit of CI can be compromised if the CN is absent or hypoplastic. However, successful implantation outcomes indicate that children can benefit from CI despite having CND. Therefore, many centers continue to offer CIs to children with CND with the consideration that some CN fibers may be present despite not being visualized by MRI (19). The present study showed that the auditory and speech abilities of children with normal CN and CND improved over time with CI use. The auditory abilities of children with a normal CN were higher than those of patients in the four CND groups starting at 6 months after CI, and this difference further increased at 12 and 24 months. Additionally, the speech abilities of children with a normal CN were higher than those of patients in the four CND groups starting at 12 months after CI use, and this difference also increased at 24 months. However, neither the auditory nor speech abilities of patients in the four CND groups differed between each group at any time point. The detailed CAP scores ranged from 3 to 5 after at least 2 years of CI use, and more than 80% of CI recipients with CND achieved CAP scores of 4 or 5, indicating that most CI recipients could understand some words and common phrases. However, none of the children in any CND group achieved CAP scores of 6 or 7, suggesting that none of them could understand daily conversations or use the telephone, in contrast to CI recipients with a normal CN in whom more than half of the CI recipients achieved CAP scores of 6 or 7. This study suggested that although the auditory and speech performance of children with CND improved over time, they were still worse than those of children with normal CN. This result was consistent with those of previous studies related to the outcomes of CI in patients with CND, which showed that individuals with CND, especially those in whom the CN was not visualized by MRI, still benefited from CI (8–13, 20, 21). This means that the absence of the CN according to MRI did not always represent the absence of the anatomical CN and that some CN fibers may be present within the vestibular or facial nerve. In our study, the performance of children with CND was not related to the IAM nerve grade, which was based on the MRI findings of nerves within the IAM and the size of the CN; this finding is not in line with those of some reports (11, 12, 22), which concluded that better performance was related to more nerve bundles. The reason for this difference might be the small sample size of these studies, as many were case reports wherein statistical analysis was not conducted. In addition, the sample size in CND I and II groups was less than other groups, leading to a large dispersion in both groups that may have contributed to the non-statistically significant difference.

There are some limitations to this study. First, although the outcomes of CI were followed for 2 years, we still did not observe a plateau; therefore, auditory and speech performance may still increase over time. Second, there were many factors that affected the outcome of CI, such as preoperative and postoperative hearing aid use. However, our study provided a relatively large sample of data and performed follow-up assessments to obtain developmental trends for the outcomes of CI in children with CND, which had rarely been reported in previous studies.

## 5. Conclusion

This study analyzed the audiological test results of children with CND and compared the auditory and speech performance after CI between children with CND and those with a normal CN. The results demonstrated that children with CND can benefit from CI even if their auditory and speech performance is below those of children with a normal CN. In addition, the IAM nerve grading system, which is based on MRI findings of nerves within the IAM and the size of the CN, could not predict the outcomes of CI in children with CND. Preoperative auditory and electrophysiological examinations, which are essential for preoperative communication with patients and their families to help them establish reasonable expectations, are also important for children with CND to determine whether they should receive a CI. We still recommend that children with CND who have no absolute contraindications for CI (such as Michel malformation, atresia of IAC, or CN canal) should undergo CI initially and that ABI should be considered if the CI outcomes are poor (23, 24).

## Data availability statement

The raw data supporting the conclusions of this article will be made available by the authors, without undue reservation.

## Ethics statement

The studies involving human participants were reviewed and approved by Ethics Committee of Xijing Hospital. Written informed consent to participate in this study was provided by the participants' legal guardian/next of kin.

## Author contributions

CR: collect and analysis, data analysis, and writing original draft. YL: writing—review and editing. XF and ZX: collect data. DZ: supervision and funding acquisition. All authors contributed to the article and approved the submitted version.
